# Roles of Emerging RNA-Binding Activity of cGAS in Innate Antiviral Response

**DOI:** 10.3389/fimmu.2021.741599

**Published:** 2021-11-26

**Authors:** Yuying Ma, Xiaohui Wang, Weisheng Luo, Ji Xiao, Xiaowei Song, Yifei Wang, Hanlin Shuai, Zhe Ren, Yiliang Wang

**Affiliations:** ^1^ Guangzhou Jinan Biomedicine Research and Development Center, National Engineering Research Center of Genetic Medicine, Institute of Biomedicine, College of Life Science and Technology, Jinan University, Guangzhou, China; ^2^ Key Laboratory of Virology of Guangdong Province, Jinan University, Guangzhou, China; ^3^ Guangdong Province Key Laboratory of Bioengineering Medicine, Jinan University, Guangzhou, China; ^4^ Department of Obstetrics and Gynecology, The Fifth Affiliated Hospital of Jinan University, Heyuan, China; ^5^ State Key Laboratory of Respiratory Disease, National Clinical Research Center for Respiratory Disease, Guangzhou Institute of Respiratory Health, the First Affiliated Hospital of Guangzhou Medical University, Guangzhou, China

**Keywords:** cGAS, RNA-binding activity, RNA-binding protein, phase-separated condensates, innate antiviral response

## Abstract

cGAS, a DNA sensor in mammalian cells, catalyzes the generation of 2’-3’-cyclic AMP-GMP (cGAMP) once activated by the binding of free DNA. cGAMP can bind to STING, activating downstream TBK1-IRF-3 signaling to initiate the expression of type I interferons. Although cGAS has been considered a traditional DNA-binding protein, several lines of evidence suggest that cGAS is a potential RNA-binding protein (RBP), which is mainly supported by its interactions with RNAs, RBP partners, RNA/cGAS-phase-separations as well as its structural similarity with the dsRNA recognition receptor 2’-5’ oligoadenylate synthase. Moreover, two influential studies reported that the cGAS-like receptors (cGLRs) of fly *Drosophila melanogaster* sense RNA and control 3′-2′-cGAMP signaling. In this review, we summarize and discuss in depth recent studies that identified or implied cGAS as an RBP. We also comprehensively summarized current experimental methods and computational tools that can identify or predict RNAs that bind to cGAS. Based on these discussions, we appeal that the RNA-binding activity of cGAS cannot be ignored in the cGAS-mediated innate antiviral response. It will be important to identify RNAs that can bind and regulate the activity of cGAS in cells with or without virus infection. Our review provides novel insight into the regulation of cGAS by its RNA-binding activity and extends beyond its DNA-binding activity. Our review would be significant for understanding the precise modulation of cGAS activity, providing the foundation for the future development of drugs against cGAS-triggering autoimmune diseases such as Aicardi-Gourtières syndrome.

## Introduction

As highlighted by the current COVID-19 pandemic, viral infection poses a great threat to human health and economics (1). The elimination of viruses by the host largely depends on the immune system, including innate immunity and adaptive immunity. Innate immunity is the first line of defense against viral infection due to its timely response and broad-spectrum effect (2). The innate antiviral response mainly refers to the process of the elimination of the virus, which involves NF-κB and interferon signaling (3). Numerous pattern recognition receptors (PRRs) can recognize pathogen-associated molecular patterns (PAMPs) and damage-associated molecular patterns (DAMPs), triggering the expression of innate antiviral factors and inflammatory factors (3). Viral PAMPs mainly include viral nucleic acids such as DNA and RNA (4). Currently, the common intracellular DNA sensors include endosomal Toll-like receptor 9 (TLR9), cytosolic absent in melanoma 2 (AIM2), interferon gamma inducible protein 16 (IFI16), DNA-dependent protein kinase (DNA-PK), RNA polymerase III, and cyclic GMP-AMP synthase (cGAS) (5, 6). Recently, two novel DNA receptors, heterogeneous nuclear ribonucleoprotein A2B1 (7) and the transmembrane protein CCDC25 (8), have been identified. Among these DNA sensors, cGAS has been well characterized and widely studied (9). Once free DNA, including viral DNA and host DNA, is recognized, cGAS synthesizes the crucial second messenger cGAMP to bind and activate the downstream stimulator of interferon genes (STING). Consequently, TANK1 binding kinase 1 (TBK1)-interferon regulatory factor-3 (IRF-3) signaling is activated to initiate the expression of type I interferons (I-IFNs), establishing the innate antiviral response (10). The absence of cGAS leads to a serious deficiency of immune defense against a series of viruses (11). Indeed, cGAS-STING is a conserved and primordial defense pathway (10, 12, 13). cGAS also plays a key role in the regulation of autoimmune diseases (14, 15) and tumor immunity (16).

Although cGAS is traditionally recognized as a DNA-binding protein (17), numerous studies have suggested the RNA-binding activity of cGAS. cGAS also plays a redundant or essential role in mediating the defense against infection with RNA viruses, such as West Nile virus (WNV) (18), dengue virus (DENV) (19), and murine norovirus (MNV) (20). Moreover, numerous studies have implied the potential of RNA-binding activity of cGAS and its involvement in cGAS-mediated innate antiviral response, which were supported by the direct binding of RNA to cGAS, the RNA-binding protein (RBP) partners of cGAS, and the structural similarity of cGAS with dsRNA recognition receptor 2’-5’ oligoadenylate synthase (OAS) (21–25). In addition, cGAS undergoes a variety of post-translational modifications in the process of performing its function (26), which partially depend on RBP modifiers. Furthermore, considering the role of noncoding RNAs and RNA-related metabolic enzymes in the DNA virus-activated innate antiviral response supported by clinical studies (23, 27, 28), we cannot ignore the RNA-binding potential of cGAS in the initiation of the innate antiviral response in addition to the traditionally recognized DNA-binding activity.

Herein, we highlighted recent studies supporting or implying the RNA-binding activity of cGAS. Simultaneously, we discussed the subcellular location of cGAS based on the reported membrane, cytosol, and nucleus localization. Moreover, we summarized current experimental and computational methods that can identify or predict the RNA interactors of cGAS. We used bioinformatics tools to analyze the interaction propensity between mouse- or human-derived cGAS and RNA. This review provides novel insight into the noncanonical regulatory manner of cGAS activity in the innate antiviral response.

## cGAS-STING Defense Pathway

**Figure 1:**
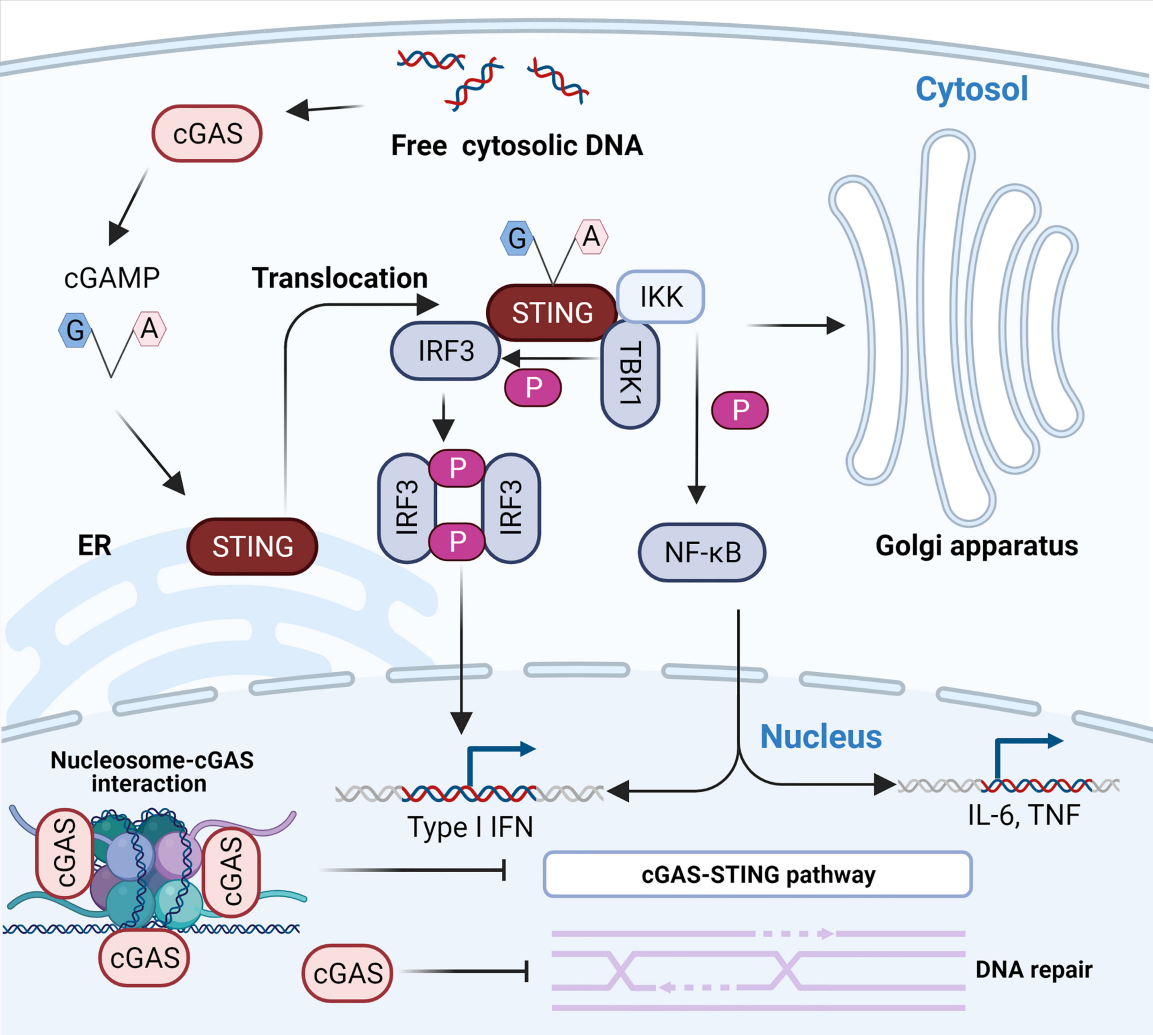
The cytosolic DNA-activated cGAS-STING pathway. The recognition of free dsDNA in the cytoplasm by cGAS activates the production of 2’-3’-cGAMP, a natural ligand of ER-resident STING. The binding of 2’-3’-cGAMP to STING results in its translocation to the ER-Golgi intermediate compartment (ERGIC) and the Golgi apparatus. The relocated STING activates TBK1 and IKK. First, activated TBK1 phosphorylates STING, which consequently recruits and phosphorylates IRF3. The phosphorylated activated IRF3 dimerizes and enters the nucleus to initiate transcription of type I IFN. In addition, activated IKK results in the activation and nuclear transport of NF-κB to induce the expression of type I IFN and inflammatory cytokines such as TNF and IL-6. The interaction between cGAS and nucleosomes prevents the activation of cGAS. Notably, nuclear cGAS suppresses homologous recombination and promotes tumorigenesis.

cGAS-STING signaling is the main pathway mediating immune defense against viruses containing DNA or producing DNA during their life cycle, such as DNA viruses and retroviruses (29, 30). Once it has recognized DNA, cGAS synthesizes 2’-3’-cGAMP using GTP and ATP as materials (10). 2’-3’-cGAMP acts as a second messenger to activate endoplasmic reticulum membrane (ER)-resident STING to form a dimer that is subsequently transported from the ER to the Golgi intermediate compartment and inter-Golgi (31) (**Figure 1**). In this process, STING recruits and activates TBK1 *via* the carboxyl terminus to phosphorylate IRF-3 (32–34) (**Figure 1**). Phosphorylated IRF-3 is dimerized and subsequently enters the nucleus to initiate the expression of IFN-β (35–37). STING also activates inhibitors of nuclear factor κB (IκB) kinase (IKK) that phosphorylate IκB, an inhibitor of NF-κB (32). The phosphorylated IκB protein is degraded by the ubiquitin–proteasome pathway, leading to the nuclear import of NF-κB (32, 38). Notably, IKKϵ allows for the redundant activation of NF-κB by STING (39). Nuclear transported NF-κB acts in concert with IRF-3 to drive the expression of IFN-β and inflammatory cytokines such as tumor necrosis factor (TNF), interleukin-1β (IL-1β), and interleukin-6 (IL-6) (32, 38) (**Figure 1**). Binding of IFN-β to the type I IFN receptor (IFNAR), which is composed of subunits IFNAR1 and IFNAR2, induces the Janus kinase (JAK)-signal transcription and activator of transcription (STAT) signaling pathway in an autocrine and paracrine manner, leading to the downstream expression of hundreds of antiviral host effector proteins (ISGs) (40, 41). These proteins block the completeness of the virus life cycle and assist neighboring cells in establishing their resistance to infection by targeting viral proteins and nucleic acids (42). However, the binding of DNA to cGAS does not always activate cGAS because the activation of downstream immune signaling pathways requires the stabilization of activated cGAS-DNA complexes and the formation of cGAS dimers (43). For example, short-stranded DNA can bind to cGAS, but such binding induces weak dimerization of cGAS (44). For longer DNA, the clustering oligomerization and arrangement of DNA induced by cGAS forms stable active dimer structures (43) and facilitates the formation of large-scale liquid-liquid phase-separated condensates (45). In these condensates, cGAS is highly concentrated, stabilizing the active dimer state and promoting its catalytic activity (45). In general, dsDNA activates cGAS more efficiently than ssDNA (46), and cGAS is much more sensitive for longer DNA than shorter DNA (47, 48).

Although cGAS has been well recognized in the process of defending against DNA viruses, many studies in recent years have shown that it also plays an instrumental role in the defense against RNA viruses (49). For instance, *cGas^−/−^* mice are more vulnerable to lethal WNV infection than wild-type mice (18). Consistently, another *in vitro* study showed that ablation of cGAS enhances MNV replication by regulating the transcription of ISGs (20). This may be because the lack of cGAS leads to decreased RIGI/MDA5 sensing of RNA (18). In addition, there is growing evidence that DNA leaking from mitochondria can engage cGAS in cells infected with RNA viruses, such as lymphocytic choriomeningitis virus (LCMV), dengue virus (DENV), encephalomyocarditis virus (EMCV), and influenza virus (19, 50–52). Specifically, influenza virus and EMCV stimulate mitochondrial DNA (mtDNA) release into the cytosol through their viroporin activity. Subsequently, cytosolic mtDNA promotes cGAS-STING-dependent innate antiviral immunity and confers resistance to RNA viruses (52). The resistance of cGAS to RNA viruses may not be due to viral RNA binding and activation of cGAS and most likely occurs in an indirect way, such as virus-induced leakage of mtDNA. Nevertheless, further research is urgently needed on the mechanism of action of cGAS against RNA virus infection.

## The DNA Sensor cGAS: A Cytosolic, Nuclear, or Membrane Protein?

cGAS was first reported as a cytoplasmic protein that can prevent cGAS from interacting with deoxyribonucleic acid in the nucleus or mitochondria (53). However, copious studies have recently found that cGAS is also localized at the cell membrane (54) and nucleus (53, 55). In detail, Orzalli et al. (55) first observed nuclear accumulation of cGAS in human fibroblasts and keratinocytes transfected with plasmid DNA. Liu et al. found that cGAS can translocate into the nucleus in response to DNA damage in the immortalized human fibroblast HCA2-TERT cell line and two primary human skin fibroblast cell lines and localize at the site where DNA damage occurs (56). Subsequently, numerous studies have shown that cGAS is constitutively present in the nucleus and even predominantly distributed in the nucleus of immune cells (57–59). More specifically, endogenous cGAS is almost exclusively localized to the nucleus in HeLa cells, primary bone marrow-derived macrophages (BMMs) (57), bone marrow‐differentiating monocytes (BMDMos) (58), and primary human monocyte-derived dendritic cells (DCs) (60), except for exogenously expressed GFP‐tagged human cGAS (GFP‐hcGAS) in HEK 293T cells (58).

Although cGAS is abundant in the nucleus, several studies almost simultaneously found that cGAS does not interact with nucleosome DNA but interacts with histones 2A and 2B of chromosomes and tightly anchors them to the “acidic patch” (59, 61–65) (**Figure 1**). This interaction masks one of the DNA-binding sites of cGAS and hinders the formation of active cGAS dimers, thereby preventing cGAS from interacting with its own genomic DNA (59, 61–65). Moreover, some extra regulatory mechanisms also limit cGAS activation, ensuring that cGAS is highly unresponsive to endogenous DNA (66, 67). For instance, barrier-to-autointegration factor 1 (BAF) dynamically inhibits the binding of cGAS to DNA and limits cGAS enzyme activity when cytoplasmic cGAS accumulates on chromatin around the nucleus during nuclear membrane rupture (66). Furthermore, the selective inhibition of cGAS activity during mitosis is attributed to two parallel mechanisms, including the hyperphosphorylation of cGAS at the N-terminus blocking the sensing of chromatin and inhibition of oligomerization of chromatin-bound cGAS (67). Intriguingly, Barnett et al. first pointed out that cGAS is not a cytoplasmic protein, as previously understood, but a membrane-localized protein that binds to plasma membrane lipids through its N-terminal domain (54). Membrane sequestration of the intracellular localization can keep cGAS away from endogenous DNA and help cGAS effectively detect viral DNA (54). Indeed, the N-terminal region of cGAS constitutes a major cytoplasmic retention signal that enables its detection and recognition in the cytosol (57) and fills an important role in regulating its activation (68). Different results regarding the localization of cGAS may be attributed to specific cells, advances in experimental techniques, and the specificity of cGAS antibodies (57). The future determination of cGAS localization requires specific cGAS antibodies that are screened by using *Cgas* ^-/-^ cells.

## cGAS Could Bind RNA and Recognize DNA-RNA Hybrids

**Figure 2:**
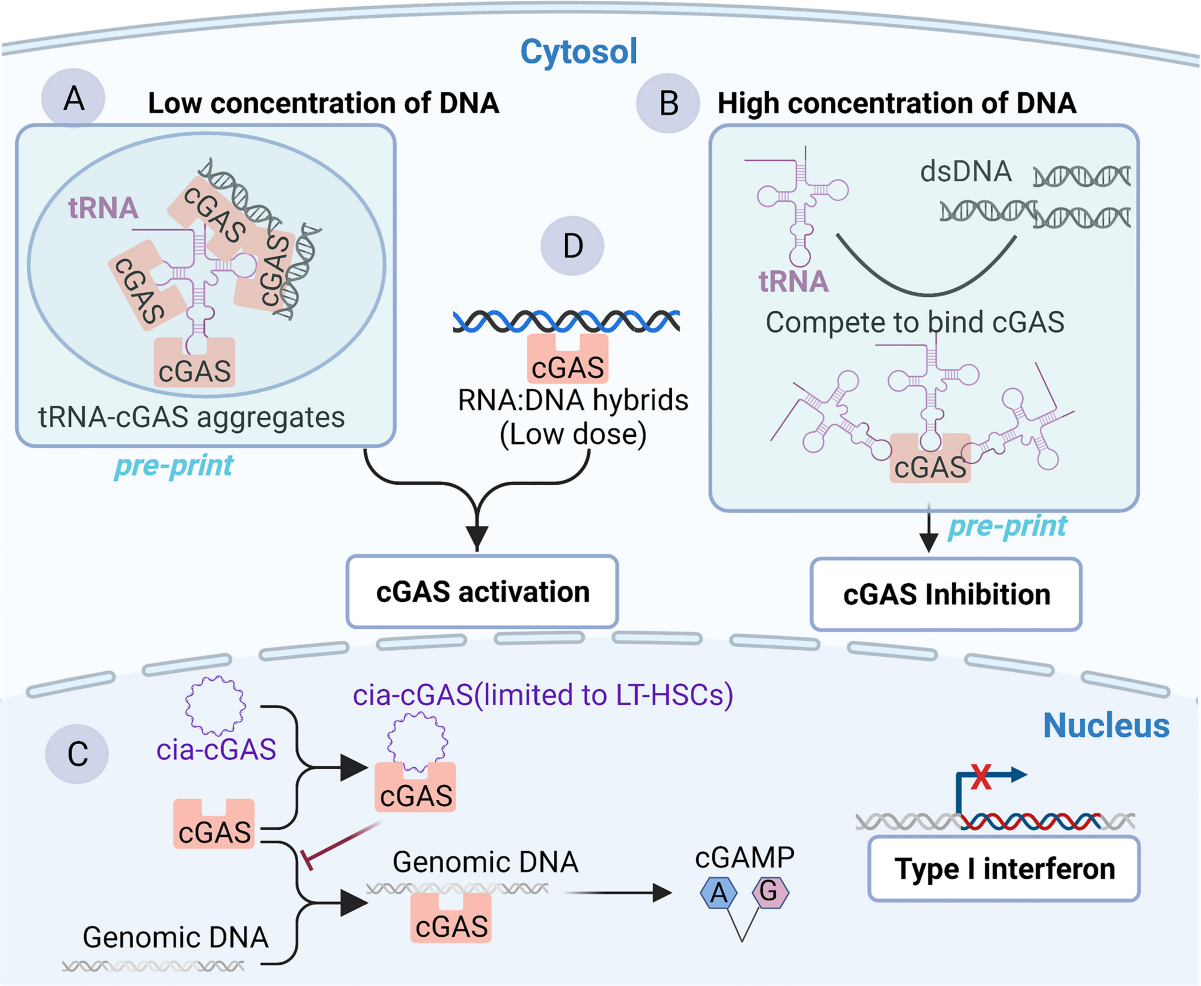
Binding of cGAS to RNA or RNA: DNA hybrids. **(A, B)** Binding of cGAS to tRNA as reported by a preprint server, which remains to be fully supported experimentally in cells. Cytoplasmic tRNA regulates cGAS activity by interfering with the formation of cGAS-containing aggregates. **(A)** In the context of low concentrations of DNA, cytoplasmic tRNA forms aggregates with cGAS, providing a platform for dsDNA-mediated cGAS activation. **(B)** In the context of a high concentration of DNA in the cytoplasm, DNA is sufficient to induce phase separation and activate cGAS. However, tRNA harbors a higher affinity than dsDNA for cGAS. Consequently, cGAS competes with dsDNA to bind cGAS and inhibit cGAS activity to avoid an excessive immune response. **(C)** Binding of cGAS to cia-cGAS. A circular RNA named cia-cGAS was highly expressed in the nucleus of LT-HSCs. Under homeostatic conditions, cia-cGAS binds cGAS in the nucleus to inhibit its binding to genomic DNA. As a consequence, cia-cGAS suppressed cGAS-mediated production of type I IFNs, thereby protecting dormant LT-HSCs from cGAS-mediated exhaustion. **(D)** RNA : DNA hybridization products can bind and activate cGAS.

Although cGAS has been widely regarded as a classical DNA-binding protein, some recent studies have uncovered or implied RNA-binding activity of cGAS. Recent *in vitro* and *in vivo* research has shown that 2′-O-methyl (2′-OMe) gapmer antisense oligonucleotides (ASOs) exhibit sequence-dependent inhibition of sensing by the RNA sensor TLR7 (69) and two major DNA sensors, cGAS and TLR9 (70). The 2’-OMe-modified RNA motifs within synthetic oligonucleotides can inhibit the recognition of cGAS DNA in a sequence-dependent manner which is independent of cGAS mRNA targeting. The 2’-OMe-modified RNA motifs compete directly for DNA binding to cGAS. Interestingly, a few ASOs can enhance cGAS sensing when used at low concentrations. The binding of these ASOs to the third DNA binding domain within cGAS was speculated to facilitate the formation of the cGAS oligomer and thus increases its enzymatic activity (70, 71). These works suggest that RNA may compete for cGAS binding to dsDNA and inhibit cGAS activity, which has also been confirmed by a preprint study (25). The test tube assay showed that RNAs compete for binding to cGAS with dsDNA, promote the formation of phase separations, enhance cGAS activity when the dsDNA concentration is low (**Figure 2A** **)** and inhibit cGAS activity when the dsDNA concentration is high (**Figure 2B**) (25). Indeed, the concentration of RNA (both tRNA and mRNA) is much higher than that of DNA in the cytoplasm (72–74) under normal conditions, which is also established under the condition of virus infection (75, 76). This makes it reasonable that RNA binds to cGAS and regulates its activity in the cytosol. However, the preliminary nature of this work must be noted: this conclusion remains to be fully supported experimentally in cells because endogenous RNAs (such as tRNA) can modulate the sensing of DNA by cGAS through the modulation of phase separations. In the absence of dsDNA, human cGAS preferentially binds to intracellular tRNA to form phase-separated droplets in the cytoplasm *via* an unidentified mechanism, ensuring a nonactivated state of cGAS; when exposed to dsDNA, the tRNAs bound by cGAS in the phase-separated particles are gradually replaced by dsDNAs due to multivalent interactions between the DNA-binding domain within cGAS and DNA (25, 45).

Another study reported the RNA-binding activity of cGAS. Covalently closed single-stranded circular RNAs (circRNAs), a class of noncoding RNAs, are characterized by a covalent bond linking the 3’ and 5’ ends during the process of RNA splicing (77, 78). cia-cGAS, an exonic circular RNA containing a paired stem region, showed a strong affinity with the DNA binding domains within cGAS. In detail, in both human and murine long-term hematopoietic stem cells (LT-HSCs), cia-cGAS binds cGAS and inhibits the binding of self-DNA to cGAS, thereby reducing the production of I-IFNs to avoid the exhaustion of HSCs to maintain the resting state of HSCs (**Figure 2C**) (22). cia-cGAS is also expressed in several tissues. Importantly, the inhibitory effect of cia-cGAS on cGAS activity also manifested in tissues expressing cia-cGAS (22). Therefore, the cia-cGAS-mediated inhibition of cGAS activity may exist in cell types but is not limited to LT-HSCs (22). However, whether cia-cGAS can bind to cGAS under viral infection conditions and participate in the regulation of antiviral natural immune responses needs to be further investigated. Of note, it has been shown earlier that cGAS can also bind to dsRNA with an affinity comparable to that of binding dsDNA, while dsRNA does not activate cGAS (21). As mentioned earlier in the article, cGAS binds to DNA in a sequence-independent manner, which is mainly associated with the length and structures of DNA (21). However, for RNA, it is clear that short synthetic oligos can compete with DNA to bind cGAS in a sequence-dependent manner (70, 79), suggesting that it is the sequence rather than the length of the RNA that may be important for the DNA binding activity of cGAS. In summary, a series of RNAs, such as synthetic oligos (70), tRNA (25), and circRNA (22), which all belong to ssRNA, can bind to cGAS and regulate its activity, whereas dsRNA can bind cGAS but does not affect the activity of cGAS (21). These results indicate that ssRNA may be able to influence cGAS activity to a greater extent than dsRNA.

cGAS can also recognize synthetic RNA : DNA hybrids and activate downstream STING-TBK1-IRF-3 signaling to initiate the expression of I-IFNs in MAVS-knockout THP-1 cells (**Figure 2C**) (80). *In vitro* studies also showed that recombinant cGAS could produce cGAMP in the presence of RNA : DNA hybrids (**Figure 2D**) (80). Moreover, although synthetic heterodimers were used in the experiments, natural RNA : DNA heterodimers can be detected from the intracellular replication cycle of retroviruses and DNA viruses such as *Herpesviridae* (81, 82). Therefore, there may be aberrant recognition of endogenous nucleic acids by cGAS during the life cycle of several viruses. Further studies need to be carried out based on the improvement of experimental techniques such as purification and detection of RNA : DNA hybrids (82, 83). Notably, the dose of cytoplasmic RNA : DNA hybrids that can induce potent antiviral immune responses by binding and activating cGAS is 10-fold less than that of dsDNA (80), suggesting that RNA/DNA hybrids are potent activators of cGAS. Alternatively, there is a study implying that cGAS may bind to RNA. Briefly, following exogenous DNA and KSHV infection, cGAS binds to the ribonucleoprotein complex HDP-RNP (HEXIM1-DNA-PK-paraspeckle) containing lncRNA NEAT1, leading to the remodeling of HDP-RNP and subsequent release of the paraspeckle protein from this complex and the recruitment of STING (84). As a consequence, downstream TBK1-IRF-3 signaling is activated. This implied that lncRNA NEAT1 may bind to cGAS and serve a regulatory function in the cGAS-mediated innate antiviral response (84). However, it will be important to address in greater detail whether cGAS can interact with NEAT1 directly, as this study did not investigate the interaction between cGAS and NEAT1 (84). Furthermore, as mentioned above, the subcellular localization of cGAS may also prompt its interaction with RNA. The distribution of cGAS in the cytosol may be involved in RNA-related physiological functions in different membrane compartments, such as stress granules (SGs) (85), liquid-liquid phase, or separated aggregates (54). Given that RNAs are first produced in the nucleus and that an RNA-filling environment is also formed in the nucleus, there may be some nuclear RNAs that regulate the activity of cGAS in the nucleus and affect the immune response: of note is the example of cia-cGAS (22). Collectively, RNA may be a crucial medium regulating the activity of cGAS by competitively binding to dsDNA rather than directly activating cGAS, ensuring precise recognition of intracellular free dsDNA to avoid immune overload.

## The RBP Interactors of cGAS

**Figure 3:**
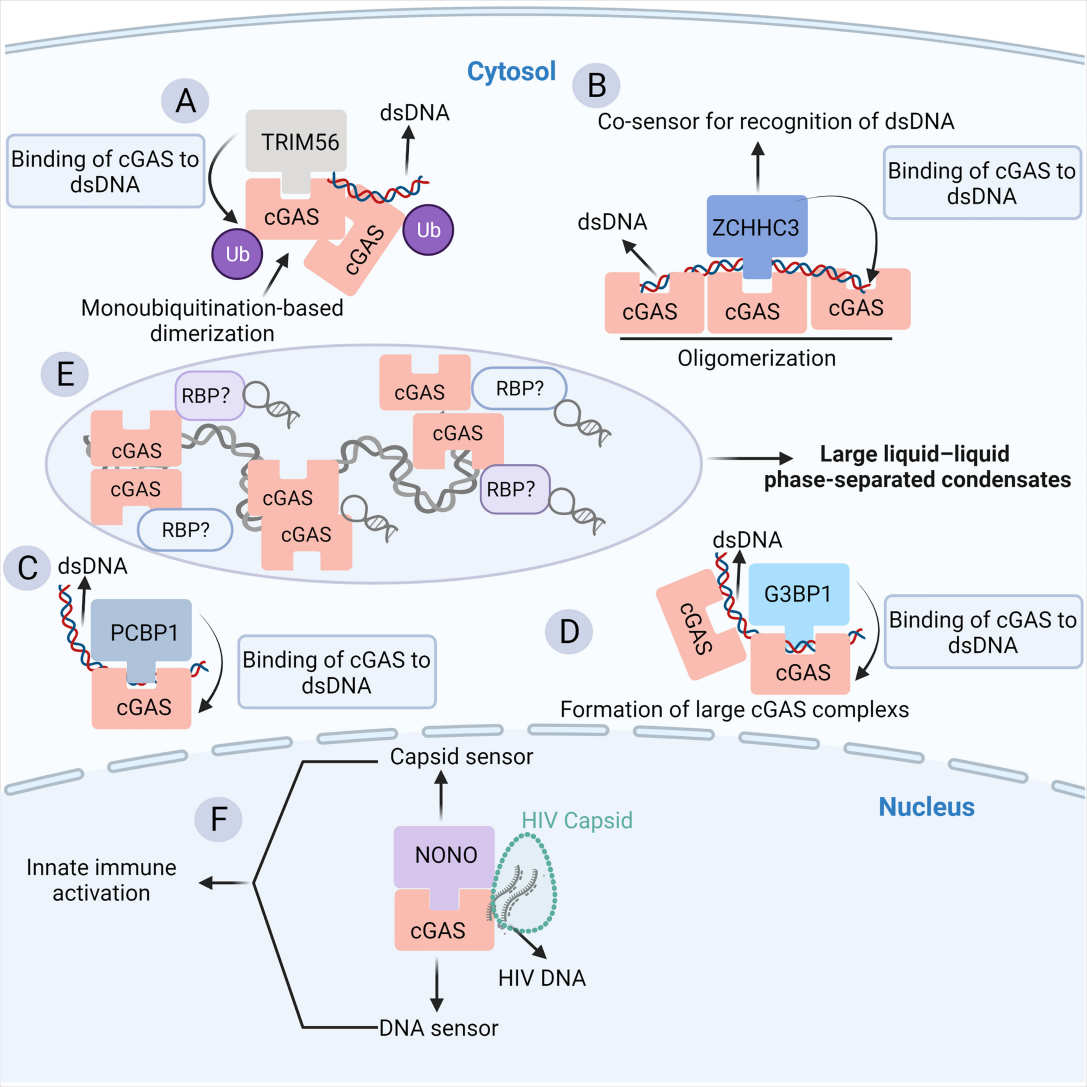
The RBP partners of cGAS. **(A)** The binding of TRIM56 to cGAS induces the Lys335 monoubiquitylation of cGAS, thereby increasing the dimerization and DNA-binding activity of cGAS. **(B)** ZCCHC3 acts as a co-sensor for the recognition of dsDNA by cGAS. Briefly, ZCCHC3 binds to dsDNA and interacts with cGAS in the cytoplasm, enhancing the binding of cGAS to dsDNA and the formation of a large cGAS complex. **(C)** PCBP1 is a critical regulator of DNA recognition by cGAS. PCBP1 was recruited to cGAS in a viral infection-dependent manner. PCBP1 bound to DNA and enhanced cGAS binding to dsDNA. **(D)** G3BP1 physically interacts with and primes cGAS for efficient activation. G3BP1 enhanced the DNA binding of cGAS by promoting the formation of large cGAS complexes. **(E)** The binding of cytosolic dsDNA to cGAS induces a robust phase transition to liquid-like droplets, which are considered as microreactors with concentrated RNA and RBPs, suggesting a potential link between cGAS and RBPs. **(F)** NONO is an essential sensor of the HIV capsid in the nucleus. NONO forms a complex with cGAS in the nucleus. Detection of the nuclear viral capsid by NONO promotes the recognition of DNA by cGAS.

In addition to the cGAS-RNA interaction, convincing evidence indicates that the binding of some classical and emerging RBPs to cGAS prompts the pivotal role of the RNA-binding activity of cGAS in the innate antiviral response. In particular, cGAS is subjected to numerous post-translational modifications to maintain immune homeostasis (26). The post-translational modifications (PTMs) that cGAS undergoes include proteasomal breakdown, acetylation, glutamylation, ubiquitination, SUMOylation, and phosphorylation (26). Most of the factors involved are RBPs, implying a strong link between cGAS and RNA. Tripartite motif (TRIM) family proteins, as multifunctional ubiquitin E3 ligases, play a central role in host defense against viral infection, which is achieved partially by regulating the PTMs of cGAS (86). TRIM56, a recently identified RBP (87, 88), binds to ZIKV RNA in infected cells and acts as a restriction factor of ZIKV (89). The binding of TRIM56 to cGAS in the cytosol induces monoubiquitylation-based dimerization of cGAS, which is important for sensing dsDNA by cGAS (24) (**Figure 3A**). Human TRIM14 recruits USP14 to cleave K48-linked cGAS ubiquitination at the K414 site, disrupting cGAS-p62 interactions to stabilize cGAS (90, 91). In addition, other classical RBPs, such as zinc finger (ZnF) CCHC-type containing 3 (ZCCHC3) (**Figure 3B**) (92) and poly (C)-binding protein 1 (PCBP1) (**Figure 3C**) (93), can bind cGAS in the cytoplasm as a cofactor to enhance the binding of cGAS to DNA, enhancing downstream antiviral natural immune responses (92, 94, 95).

In particular, G3BP1, a core component of SGs, is a classical RBP (85). Additionally, the G3BP1-RNA interaction network is a key node in the formation of SG particles (96–98). G3BP1 can bind and promote the binding of cGAS to DNA, which in turn enhances downstream I-IFN signaling (**Figure 3D**) (99). The close relationship between G3BP1, SG, and cGAS implies that the abundant RNA and RBPs inside SG particles may be crucial regulators of cGAS activity (**Figure 3E**). Indeed, SGs are produced in response to stress, such as virus infection. In such a context, RNAs, a large number of RBPs, and other molecules are rapidly condensed into cytoplasmic SGs by phase separation (100). Moreover, it has been shown that cGAS occurs in this liquid-liquid phase separation structure (53), indicating that cGAS may interact with related components of SGs (**Figure 3E**). NONO is another multifunctional RBP implicated in transcription, splicing, DNA damage response, circadian rhythm, and neuronal development (101). NONO can recognize the HIV capsid and facilitate the binding of cGAS to HIV DNA, which is required for cGAS activation (**Figure 3F**) (101, 102). A recent study also showed that the disruption of the ribosome-associated protein quality control (RQC) pathway under stressful conditions such as viral infection, which detects and resolves ribosome collision during translation, results in cGAS-dependent ISG expression and causes relocalization of cGAS from the nucleus to the cytosol (103). Nevertheless, the RBP interactors of cGAS only suggest the potential RNA-binding activity of cGAS. In the future, it will be important to uncover the RNA interactors of cGAS in more detail by using RNA pull-down and RNA-immunoprecipitation experiments.

## Structure-Based Analysis of the RNA-Binding Activity of cGAS

Typical RBPs are defined by the presence of RNA-binding domains (RBDs), such as hnRNP K homology domains, RNA recognition motifs, dsRNA-binding domains, and ZnF domains (104–106). However, many proteins lacking conventional RBDs or even DNA-binding proteins have been gradually recognized as factors with RNA-binding activity (23, 107–109). From the perspective of protein structure, cGAS consists of a disordered, positively charged N-terminal domain, a central nucleotidyltransferase (NTase) structural domain, a zinc-finger domain, and a C-terminal Mab-21 homologous structural domain (17, 110). The unique zinc-finger domain might be the decisive structural domain for its DNA binding activity. Interestingly, cGAS shares striking structural and functional similarities with the dsRNA recognition receptor 2′-5′-OAS (21, 111). In detail, both cGAS and 2′-5′-OAS are template-independent NTases. Moreover, cGAS and 2′-5′-OAS share overall folding structure, characterization of several active sites, the arrangement of blade 1 and blade 2, and even the 3D structural elements of the nucleic acid binding site (49, 112). Furthermore, the activation of both cGAS and 2′-5′-OAS is associated with the length of nucleic acids (111). From the perspective of function, cGAS and 2′-5′-OAS can be activated by similar double-stranded nucleic acid-inducible structural switches (21, 111). Once activated, cGAS and 2′-5′-OAS can generate a unique 2’-5’ phosphodiester-linked second messenger of nucleotides to initiate the relevant IFN-I immune pathway (21, 111). Based on evolutionary terms, the structures of cGAS are highly similar to those of 2′-5′-OAS (21, 46), especially the shared structure fold (21, 46), suggesting a common evolutionary ancestral origin as a structurally related but functionally distinct family of cytoplasmic nucleic acid sensors. Indeed, some scholars have merged cGAS and 2′-5′-OAS into a new family of catalytic OAS-like second messenger receptors (OLRs) (46). Notably, two recent studies have identified a class of cGAS-like receptors (cGLRs) in *Drosophila* that share a bi-lobed architecture, a caged nucleotidyltransferase core, a Gly-[Gly/Ser] activation loop, and a putative catalytic triad with cGAS (113). cGLRs recognize dsRNA and activate the production of the nucleotide product 3′-2′-cGAMP, and the signal is preferentially recognized by the *Drosophila* stimulator of interferon genes (dSTING), which exerts antiviral immunity in conjunction with the NF-κB pathway (114). It can be speculated that this conserved nucleic acid recognition structure is of great significance in the evolution of postnatal 2′-5′-OAS, cGAS, and cGLR animal proteins.

**Figure 4:**
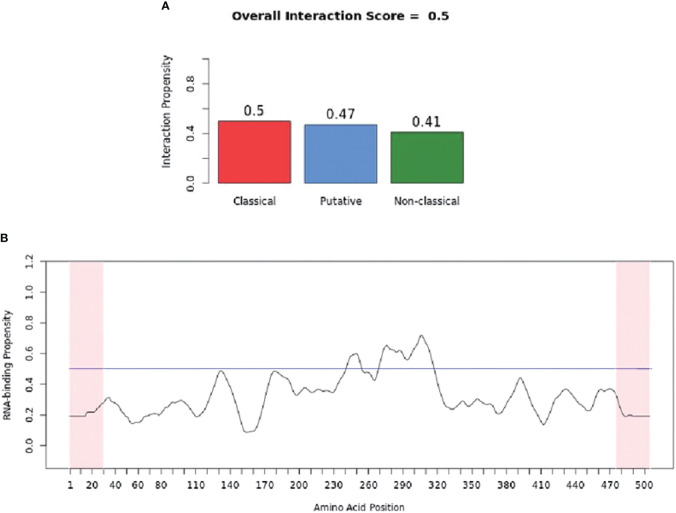
RNA-binding ability of murine-cGAS determined by catRAPID signature. **(A)** The propensity of murine cGAS for classical (0.5), putative (0.47), and nonclassical (0.41) RBPs. The overall interaction score (0.5) for murine cGAS as an RBP was also present. **(B)** The profile shows protein regions and their propensity to interact with RNA. All results were predicted by using catRAPID.

**Figure 5:**
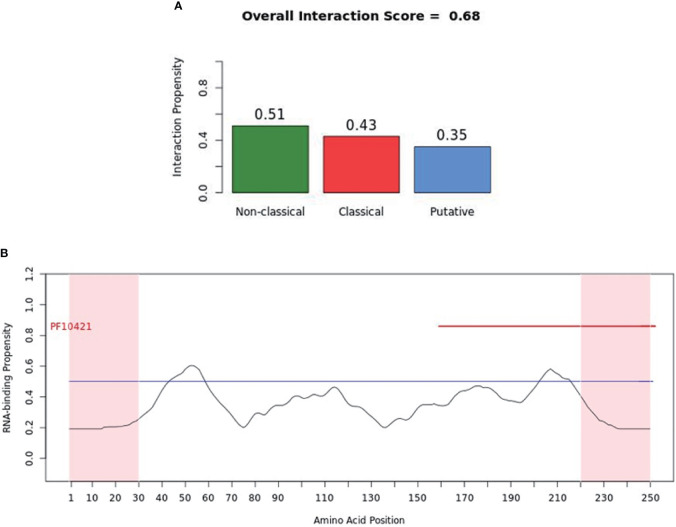
RNA-binding ability of murine-2’-5’-OAS1 predicted by catRAPID signature. **(A)** The propensity of murine-2’-5’-OAS1 for the nonclassical (0.51), classical (0.43) and putative (0.35) RBPs. The overall interaction score (0.68) for murine-2’-5’-OAS1 as an RBP was also present. **(B)** The profile shows protein regions and their propensity to interact with RNA. The catRAPID signature correctly identifies the RNA binding domain of murine-2’-5’-OAS1, which carries the region of enzymatic activity between 320 and 344 at the extreme C-terminal end (122).

catRAPID (http://service.tartaglialab.com/page/catrapid_group) is an online algorithm predicting the propensity of protein-RNA interactions based on the respective contributions of secondary structure, hydrogen bonding, and Van Der Waals interactions (115). catRAPID has been widely used to study protein-RNA interactions with high accuracy (116–120). We first used the catRAPID signature (121) to predict the RNA-binding potential of murine cGAS. The overall prediction score of mcGAS was 0.5 (**Figure 4**), reflecting potential RNA-binding activity of mcGAS. Further analysis indicated that the RNA-binding activity of cGAS was mainly attributed to amino acids 234-331 within the Mab-21 structural domain (**Figure 4**). We also analyzed the RNA binding activity of 2’-5’-OAS1 as a well-characterized RBP using the catRAPID signature, and the results indicated a higher predicted RNA binding score of 0.68 compared to cGAS (**Figure 5**). We next used catRAPID omics (123, 124) to discover the potential RNA interactors of murine cGAS (**Table 1**) and human cGAS (**Table 2**), which provides novel insight for future study of the RNA interactors of cGAS. However, the potential RNA interactors of cGAS should be determined by using relevant experimental methods. Nevertheless, these predicted results based on bioinformatic analysis further demonstrated the potential RNA-binding activity of cGAS.

**Table 1:** The RNA interactors of murine cGAS predicted by catRAPID omics (version 2.0).

	RNA ID	Gene Name	Interaction Propensity	Z-score	RBP Propensity	RNA-Binding Domains	RNA-Binding Motifs	Conserved Interactions	Ranking
protein-coding RNAs	ENSMUST00000000080	Kruppel-like Factor 6 (Klf6)	88.83	1.73	0.5	1	0	0/0	0.405431
	ENSMUST00000000291	max binding protein (Mnt)	73.94	1.34	0.5	1	0	0/0	0.389061
	ENSMUST00000000579	sex determining region Y-box 9 (Sox9)	56.82	0.89	0.5	1	0	0/0	0.37024
	ENSMUST00000000619	chloride channel, voltage-sensitive 4 (Clcn4)	74.44	1.35	0.5	1	0	0/0	0.389611
	ENSMUST00000000724	K (lysine) acetyltransferase 2B (Kat2b)	61.09	1	0.5	1	0	0/0	0.374934
long noncoding RNAs	ENSMUST00000047876	Gm10710	45.58	0.59	0.5	1	0	0/0	0.357883
	ENSMUST00000052189	B230317F23Rik	49.89	0.7	0.5	1	0	0/0	0.362621
	ENSMUST00000097612	Gm10545	49.12	0.68	0.5	1	0	0/0	0.361774
	ENSMUST00000098303	Gm9934	42.98	0.52	0.5	1	0	0/0	0.355024
	ENSMUST00000100713	Gm10384	41.97	0.49	0.5	1	0	0/0	0.353914
small noncoding RNAs	ENSMUST00000082459	Gm23627	25.98	0.07	0.49	1	0	0/0	0.333001
	ENSMUST00000082490	Gm22749	28.99	0.15	0.49	1	0	0/0	0.33631
	ENSMUST00000082508	Gm26225	27.07	0.1	0.49	1	0	0/0	0.3342
	ENSMUST00000082509	Gm26226	36.2	0.34	0.49	1	0	0/0	0.344237
	ENSMUST00000082534	Gm25081	25.39	0.06	0.49	1	0	0/0	0.332353

Table summarization of the top 5 predicted coding-RNA interactors (RNA ID and Gene Name) of murine-cGAS by catRAPID omics (version 2.0). The table shows Z-scores (interaction propensity normalization relative to experimental cases), discriminative ability (relative to training sets), interaction strength (enrichment relative to random interactions), the presence of RNA-binding domains, and RNA motifs. RNAs were ranked by the score, which is the sum of three individual values: 1) catRAPID normalized propensity, 2) RBP propensity and 3) presence of known RNA-binding motifs. The full score is 1.

**Table 2:** The RNA interactors of human cGAS predicted by catRAPID omics (version 2.0).

	RNA ID	Gene Name	Interaction Propensity	Z-score	RBP Propensity	RNA-Binding Domains	RNA-Binding Motifs	Conserved Interactions	Ranking
protein-coding RNAs	ENST00000078429	G protein subunit alpha 11 (GNA11)	57.38	0.9	0.35	1	0	0/0	0.320855
	ENST00000169298	ST6 beta-galactoside alpha-2,6-sialyltransferase 1 (ST6GAL1)	56.82	0.89	0.35	1	0	0/0	0.32024
	ENST00000170168	RNA exonuclease 1 homolog (REXO1)	59.47	0.96	0.35	1	0	0/0	0.323153
	ENST00000174618	MAX network transcriptional repressor (MNT)	60.79	0.99	0.35	1	0	0/0	0.324604
	ENST00000193322	osteoclastogenesis associated transmembrane protein 1 (OSTM1)	54.06	0.81	0.35	1	0	0/0	0.317205
long noncoding RNAs	ENST00000242109	KIAA0087	24.68	0.04	0.35	1	0	0/0	0.284905
	ENST00000309874	AC020659.1	19.83	-0.09	0.35	1	0	0/0	0.279573
	ENST00000316786	C9orf106	21.42	-0.05	0.35	1	0	0/0	0.281321
	ENST00000325390	AC018865.2	20.01	-0.09	0.35	1	0	0/0	0.279771
	ENST00000329618	B3GALT5-AS1	20.99	-0.06	0.35	1	0	0/0	0.280849
small noncoding RNAs	ENST00000347538	MIR133A2	4.53	-0.49	0.35	1	0	0/0	0.262753
	ENST00000362117	MIR16-2	6.2	-0.45	0.35	1	0	0/0	0.264589
	ENST00000362317	MIR365B	7.6	-0.41	0.35	1	0	0/0	0.266128
	ENST00000362349	RNU6-500P	6.08	-0.45	0.35	1	0	0/0	0.264457
	ENST00000362356	RNU6-50P	5.19	-0.48	0.35	1	0	0/0	0.263478

Table summarization of the top 5 predicted coding-RNA interactors (RNA ID and Gene Name) of murine-cGAS by catRAPID omics (version 2.0). The table shows Z-scores (interaction propensity normalization relative to experimental cases), discriminative ability (relative to training sets), interaction strength (enrichment relative to random interactions), the presence of RNA-binding domains, and RNA motifs. RNAs were ranked by the score, which is the sum of three individual values: 1) catRAPID normalized propensity, 2) RBP propensity and 3) presence of known RNA-binding motifs. The full score is 1.

## Computational and Experimental Methods for Detecting or Predicting cGAS-RNA Interactions

Numerous experimental methods and computational methods have been devised to reveal RNA-protein interactions (125, 126). We comprehensively summarized the current experimental methods (**Table 3**) and computational tools (**Table 4**) for studying RNA-protein interactions as well as their respective advantages and disadvantages. These methods would be beneficial for us to identify RNAs that bind to cGAS and reveal the RNA-binding activity of cGAS and its regulatory function in the antiviral immune response. The experimental methods can be further classified according to *cells* and *test tubes* in terms of experimental conditions. The latter mainly include electrophoretic mobility shift assay (EMSA), RNA pull-down, systematic evolution of ligands by exponential enrichment (SELEX), RNAcompete, and RBNS (**Table 3**). The *in cells* assays mainly include RNA binding protein immunoprecipitation (RIP) and crosslinking immunoprecipitation (CLIP) assays (**Table 3**). In fact, the concentrations of RBPs and RNAs used in test tubes are much higher than those levels in a physiological context (144). Furthermore, the artificially created *in vitro* environment is pure and susceptible to technical influence (145). Therefore, the results of the test tube assays cannot represent the true physiological mechanisms of interactions *in vivo*.

**Table 3:** Experimental methods for uncovering the interaction between proteins and RNAs.

Classes	Methods	Advantages	Disadvantages	References
In the test tube	EMSA	Separation of numerous types of complexes, such as monomer and dimer; Works well with crude cell extracts.	Low-throughput; Failure of detecting binding sites.	(125, 127)
	RNA pull-down	A simple protocol; Enrichment of low-abundance RBPs.	Failure of confirming direct or indirect interaction; Failure of forming RNA-protein interactions that only occur in vitro under non-physiological conditions.	(125, 128)
	SELEX	Direct interaction of the oligonucleotides with the target is closely application-oriented; Independence of in-depth knowledge regarding the respective target for aptamer selection.	No standardized aptamer selection protocol for target.	(129–132)
	RNAcompete	Accurate estimation of the relative preference for a large numbers of individual sequences; Querying preferences are available for structured RNA; Time-saving.	Limited size of the current RNA pool for the represented combinations of sequence and structure.	(125, 133)
	RBNS	Accurate estimation of dissociation constants of RBP-RNA complex; A more reliable prediction of RNA folding and a better identification of the binding structural determinants than RNAcompete.	N/A	(125, 134, 135)
In cells	RIP Assay	A simple and standard protocol; Preserves the intracellular native complexes.	Failure of confirmation of direct or indirect interaction between RNA and protein; Failure of determination of the precise site within the RNA interacting protein; Poor resolution; Antibody-consuming.	(125, 136)
	CLIP	A sensitive determination of binding sites.	Low abundance of RNA-ribonucleoprotein complexes; Potentially inefficient library preparation; Large amounts of raw material required.	(108, 125, 137)
	PIP-seq	A simultaneous view of the global landscapes of both RNA secondary structure and RNA-protein interactions.	High concentrations of structure-specific RNases.	(138)

EMSA, Electrophoretic Mobility Shift Assay; SELEX, Systematic Evolution of Ligands by Exponential Enrichment; RBNS; RNA Bind-n-Seq; RIP, RNA Immunoprecipitation; CLIP, Cross-linked Immunoprecipitation; PIP-seq, Protein Interaction Profile Sequencing; N/A, not applicable.

**Table 4:** Computational tools for the prediction of RNA-protein interactions.

Methods	Advantages	Disadvantages	Links	References
catRAPID	Outperforms other algorithms in the identification of RBPs and detection of non-classical RNA-binding regions.	Spurious binding may occur.	http://s.tartaglialab.com/page/catrapid_group	(115, 121)
lncPro	Time-saving.	Limited ability of computational prediction of RNA secondary structure for lncRNAs.	bioinfo.bjmu.edu.cn/lncpro/	(139)
RNAcontext	A more accurate elucidation of RBP-specific sequence and structural preferences.	Misleading results will be produced for those RBPs with non-trivial structural preferences.	http://www.cs.toronto.edu/~hilal/rnacontext/	(140)
RPI-Pred	A comprehensive understanding of PRIs based on the sequence features and the high-order structures of both proteins and RNAs.	Limited to prediction of ncRNA-Protein interaction.	http://ctsb.is.wfubmc.edu/projects/rpi-pred.	(141)
PRIPU	Employs SVM for predicting PRIs using only positive and unlabeled examples based on proposing a new performance measure called EPR; Outperforms existing methods and predicts unknown PRIs.	N/A	http://admis.fudan.edu.cn/projects/pripu.htm	(142)
RPISeq	Reliable prediction based on sequence.	Requirement for larger and more diverse experimental datasets.	RNA-Protein Interaction Prediction (RPISeq) (iastate.edu)	(143)

RBPs, RNA-binding proteins; SVM, biased-support vector machine; lncRNA, long noncoding RNA; PRIS, Protein-RNA interactions; EPR, explicit positive recall; N/A, not applicable.

Although experimental methods could provide convincing evidence supporting the RNA-protein interaction, most of the experimental methods are technically challenging and time-consuming (**Table 3**). In contrast, the computational approaches are simple, fast, and time-saving and can be used as ideal amendments to the experimental methodology (125, 146–149) (**Table 4**). The growing numbers of public experimental data also offer the possibility to train computational models for inferring new interactions. Most of these computational methods for predicting RNA-protein interactions (such as catRAPID, lncPro, and RNAcontext) calculate their propensity based on the physicochemical properties of peptides and nucleotide chains (e.g., propensity for hydrogen bonding, van der Waals interactions, and secondary structure) (125) (**Table 4**). On the basis of these principles, there are also methods such as RPI-Pred and PRIPU that further introduced statistical and machine learning algorithms, including vector machines, in the prediction of RNA-protein interactions (141, 142) (**Table 4**).

## Conclusions and Future Perspectives

In this review, we highlighted the RNA-binding activity of cGAS based on the RNA interactors of cGAS, the numerous RBP partners of cGAS, and its structural similarity with 2′-5′-OAS. We also elucidated and discussed current experimental methods and computational tools that can be used for exploring and calculating cGAS-RNA interactions. These clues indicated that the RNA-binding activity of cGAS may play an important role in the innate antiviral response, which needs to be further confirmed in the future. Although cGAS showed potential RNA-binding activity, it should be noted that the RNA binding activity of cGAS is different from that of known RNA sensors, such as RIG-1 and TLR3, which mainly recognize viral RNA (23). In contrast, known dsRNA interactors cannot activate cGAS (21). Furthermore, given that the known interacting RNAs of cGAS are not derived from a virus (21, 22) and that cGAS plays a redundant role in recognizing RNA viruses (49, 150), the RNA-binding activity of cGAS may largely affect the DNA-binding activity of cGAS but does not mean that cGAS can recognize viral RNA or RNA interactors which directly activate cGAS. In fact, since the discovery of cGAS as a novel receptor for dsDNA, researchers have used a variety of physical and biochemical methods to explore the mechanism of action of dsDNA recognition by cGAS. However, numerous reports have also begun to prove the RNA-binding activity of cGAS. Of note, no studies have proven that binding RNA could act as a PAMP to directly activate cGAS. The RNA-binding ability of cGAS in the innate antiviral response represents a scientific question that urgently needs to be resolved. In summary, the RNA interactor of cGAS could largely modulate cGAS function rather than activate it. Nevertheless, we cannot ignore the role of the RNA-binding activity of cGAS in the innate antiviral response, although cGAS is recognized as a classical DNA-binding protein. Moreover, cGAS can be activated by self-DNA, which triggers the development of autoimmune diseases such as Aicardi-Gourtières syndrome (151). Therefore, it is important to address the RNA-binding activity of cGAS in the innate antiviral response in more detail to understand the precise modulation of cGAS activity. Our review provides a comprehensive understanding of the regulation of cGAS activity, which extends beyond the DNA-binding activity of cGAS. Our review is significant for understanding cGAS signaling.

## Author Contributions

YM, XW, HS, YilW, WS, JX, and WL: conception and design, collection and/or assembly of references, data analysis and interpretation, and manuscript writing; HS, ZR, YifW, and YilW: final interpretation and helpful discussion of the manuscript. All authors read and approved the final manuscript and agreed to this publication. YM and XW contributed equally to this article.

## Funding

This work was supported by grants from the National Natural Science Foundation of China (Nos. 81872908 and 82072274), Key Laboratory of Virology of Guangdong Province, Guangdong Provincial Natural Science Foundation Project (No. 2019A1515010046), Study on the mechanism of estrogen promoting the repair of urethral function in the treatment of female stress urinary incontinence with hUCMSCs (No. 2021A1515011221), and Guangdong Modern Agricultural Industrial Technology System Innovation Team (No. 2017YFC1701100).

## Conflict of Interest

The authors declare that the research was conducted in the absence of any commercial or financial relationships that could be construed as a potential conflict of interest.

## Publisher’s Note

All claims expressed in this article are solely those of the authors and do not necessarily represent those of their affiliated organizations, or those of the publisher, the editors and the reviewers. Any product that may be evaluated in this article, or claim that may be made by its manufacturer, is not guaranteed or endorsed by the publisher.

## References

[1] Luo W Huang L Wang X Ma Y Xiao J Song X. SARS-CoV-2 Infection Activates a Subset of Intrinsic Pathways to Inhibit Type I Interferons *In Vitro* and In Vivo. Int J Med Sci (2021) 18(12):2561. doi: 10.7150/ijms.56630 34104087 PMC8176179

[2] Ike AC Onu CJ Ononugbo CM Reward EE Muo SO. Immune Response to Herpes Simplex Virus Infection and Vaccine Development. Vaccines (Basel) (2020) 8(2):302. doi: 10.3390/vaccines8020302 PMC7350219 32545507

[3] Ivashkiv LB Donlin LT. Regulation of Type I Interferon Responses. Nat Rev Immunol (2014) 14(1):36–49. doi: 10.1038/nri3581 24362405 PMC4084561

[4] Unterholzner L Bowie AG. The Interplay Between Viruses and Innate Immune Signaling: Recent Insights and Therapeutic Opportunities. Biochem Pharmacol (2008) 75(3):589–602. doi: 10.1016/j.bcp.2007.07.043 17868652

[5] Zhao J Qin C Liu Y Rao Y Feng P. Herpes Simplex Virus and Pattern Recognition Receptors: An Arms Race. Front Immunol (2020) 11:613799. doi: 10.3389/fimmu.2020.613799 33584700 PMC7878388

[6] Hu MM Shu HB. Innate Immune Response to Cytoplasmic DNA: Mechanisms and Diseases. Annu Rev Immunol (2020) 38:79–98. doi: 10.1146/annurev-immunol-070119-115052 31800327

[7] Wang L Wen M Cao X. Nuclear Hnrnpa2b1 Initiates and Amplifies the Innate Immune Response to DNA Viruses. Science (2019) 365(6454):656–+. doi: 10.1126/science.aav0758 31320558

[8] Yang L Liu Q Zhang X Liu X Zhou B Chen J. DNA of Neutrophil Extracellular Traps Promotes Cancer Metastasis *via* CCDC25. Nature (2020) 583(7814):133–8. doi: 10.1038/s41586-020-2394-6 32528174

[9] Li XD Wu J Gao D Wang H Sun L Chen ZJ. Pivotal Roles of cGAS-cGAMP Signaling in Antiviral Defense and Immune Adjuvant Effects. Science (2013) 341(6152):1390–4. doi: 10.1126/science.1244040 PMC3863637 23989956

[10] Ma Z Damania B. The cGAS-STING Defense Pathway and Its Counteraction by Viruses. Cell Host Microbe (2016) 19(2):150–8. doi: 10.1016/j.chom.2016.01.010 PMC4755325 26867174

[11] Schoggins JW MacDuff DA Imanaka N Gainey MD Shrestha B Eitson JL. Pan-Viral Specificity of IFN-Induced Genes Reveals New Roles for cGAS in Innate Immunity. Nature (2015) 525(7567):144. doi: 10.1038/nature14555 PMC8323779 26153856

[12] Gui X Yang H Li T Tan X Shi P Li M. Autophagy Induction *via* STING Trafficking Is a Primordial Function of the cGAS Pathway. Nature (2019) 567(7747):262–6. doi: 10.1038/s41586-019-1006-9 PMC9417302 30842662

[13] Morehouse BR Govande AA Millman A Keszei AFA Lowey B Ofir G. STING Cyclic Dinucleotide Sensing Originated in Bacteria. Nature (2020) 586(7829):429–33. doi: 10.1038/s41586-020-2719-5 PMC7572726 32877915

[14] Gao D Li T Li XD Chen X Li QZ Wight-Carter M. Activation of Cyclic GMP-AMP Synthase by Self-DNA Causes Autoimmune Diseases. Proc Natl Acad Sci U S A (2015) 112(42):E5699–705. doi: 10.1073/pnas.1516465112 PMC4620884 26371324

[15] An J Durcan L Karr RM Briggs TA Rice GI Teal TH. Expression of Cyclic GMP-AMP Synthase in Patients With Systemic Lupus Erythematosus. Arthritis Rheumatol (2017) 69(4):800–7. doi: 10.1002/art.40002 27863149

[16] Chen Q Sun L Chen ZJ. Regulation and Function of the cGAS-STING Pathway of Cytosolic DNA Sensing. Nat Immunol (2016) 17(10):1142–9. doi: 10.1038/ni.3558 27648547

[17] Sun LJ Wu JX Du FH Chen X Chen ZJJ. Cyclic GMP-AMP Synthase Is a Cytosolic DNA Sensor That Activates the Type I Interferon Pathway. Science (2013) 339(6121):786–91. doi: 10.1126/science.1232458 PMC3863629 23258413

[18] Schoggins JW MacDuff DA Imanaka N Gainey MD Shrestha B Eitson JL. Pan-Viral Specificity of IFN-Induced Genes Reveals New Roles for cGAS in Innate Immunity. Nature (2014) 505(7485):691–+. doi: 10.1038/nature12862 PMC4077721 24284630

[19] Aguirre S Luthra P Sanchez-Aparicio MT Maestre AM Patel J Lamothe F. Dengue Virus NS2B Protein Targets cGAS for Degradation and Prevents Mitochondrial DNA Sensing During Infection. Nat Microbiol (2017) 2(5):17037. doi: 10.1038/nmicrobiol.2017.37 28346446 PMC7457382

[20] Yu P Miao Z Li Y Bansal R Peppelenbosch MP Pan Q. cGAS-STING Effectively Restricts Murine Norovirus Infection But Antagonizes the Antiviral Action of N-Terminus of RIG-I in Mouse Macrophages. Gut Microbes (2021) 13(1):1959839. doi: 10.1080/19490976.2021.1959839 34347572 PMC8344765

[21] Civril F Deimling T Mann CCD Ablasser A Moldt M Witte G. Structural Mechanism of Cytosolic DNA Sensing by cGAS. Nature (2013) 498(7454):332–+. doi: 10.1038/nature12305 PMC3768140 23722159

[22] Xia P Wang S Ye B Du Y Li C Xiong Z. A Circular RNA Protects Dormant Hematopoietic Stem Cells From DNA Sensor cGAS-Mediated Exhaustion. Immunity (2018) 48(4):688–701.e7. doi: 10.1016/j.immuni.2018.03.016 29625897

[23] Wang Y Wang Y Luo W Song X Huang L Xiao J. Roles of Long Non-Coding RNAs and Emerging RNA-Binding Proteins in Innate Antiviral Responses. Theranostics (2020) 10(20):9407–24. doi: 10.7150/thno.48520 PMC7415804 32802200

[24] Seo GJ Kim C Shin WJ Sklan EH Eoh H Jung JU. TRIM56-Mediated Monoubiquitination of cGAS for Cytosolic DNA Sensing. Nat Commun (2018) 9(1):613. doi: 10.1038/s41467-018-02936-3 29426904 PMC5807518

[25] Chen S Rong M Lv Y Zhu D Xiang Y. Regulation of cGAS Activity Through RNA-Mediated Phase Separation. bioRxiv (2020). doi: 10.1101/2020.09.27.316166

[26] Baker PJ De Nardo D Moghaddas F Tran LS Bachem A Nguyen T. Posttranslational Modification as a Critical Determinant of Cytoplasmic Innate Immune Recognition. Physiol Rev (2017) 97(3):1165–209. doi: 10.1152/physrev.00026.2016 28615462

[27] Zhang SY Clark NE Freije CA Pauwels E Taggart AJ Okada S. Inborn Errors of RNA Lariat Metabolism in Humans With Brainstem Viral Infection. Cell (2018) 172(5):952–65.e18. doi: 10.1016/j.cell.2018.02.019 29474921 PMC5886375

[28] Lafaille FG Harschnitz O Lee YS Zhang P Hasek ML Kerner G. Human SNORA31 Variations Impair Cortical Neuron-Intrinsic Immunity to HSV-1 and Underlie Herpes Simplex Encephalitis. Nat Med (2019) 25(12):1873–84. doi: 10.1038/s41591-019-0672-3 PMC7376819 31806906

[29] Abe T Marutani Y Shoji I. Cytosolic DNA-Sensing Immune Response and Viral Infection. Microbiol Immunol (2019) 63(2):51–64. doi: 10.1111/1348-0421.12669 30677166 PMC7168513

[30] Wu F Zhao S Yu B Chen Y Wang W Song Z-G. A New Coronavirus Associated With Human Respiratory Disease in China. Nature (2020) 579(7798):1–8. doi: 10.1038/s41586-020-2008-3 PMC7094943 32015508

[31] Motwani M Pesiridis S Fitzgerald KA. DNA Sensing by the cGAS-STING Pathway in Health and Disease. Nat Rev Genet (2019) 20(11):657–74. doi: 10.1038/s41576-019-0151-1 31358977

[32] Ishikawa H Barber GN. STING Is an Endoplasmic Reticulum Adaptor That Facilitates Innate Immune Signalling. Nature (2008) 455(7213):674–8. doi: 10.1038/nature07317 PMC2804933 18724357

[33] Ishikawa H Ma Z Barber GN. STING Regulates Intracellular DNA-Mediated, Type I Interferon-Dependent Innate Immunity. Nature (2009) 461(7265):788–92. doi: 10.1038/nature08476 PMC4664154 19776740

[34] Liu S Cai X Wu J Cong Q Chen X Li T. Phosphorylation of Innate Immune Adaptor Proteins MAVS, STING, and TRIF Induces IRF3 Activation. Science (2015) 347(6227):aaa2630. doi: 10.1126/science.aaa2630 25636800

[35] Sato M Tanaka N Hata N Oda E Taniguchi T. Involvement of the IRF Family Transcription Factor IRF-3 in Virus-Induced Activation of the IFN-Beta Gene. FEBS Lett (1998) 425(1):112–6. doi: 10.1016/S0014-5793(98)00210-5 9541017

[36] Yoneyama M Suhara W Fukuhara Y Fukuda M Nishida E Fujita T. Direct Triggering of the Type I Interferon System by Virus Infection: Activation of a Transcription Factor Complex Containing IRF-3 and CBP/P300. EMBO J (1998) 17(4):1087–95. doi: 10.1093/emboj/17.4.1087 PMC1170457 9463386

[37] Schwanke H Stempel M Brinkmann MM. Of Keeping and Tipping the Balance: Host Regulation and Viral Modulation of IRF3-Dependent IFNB1 Expression. Viruses (2020) 12(7):733. doi: 10.3390/v12070733 PMC7411613 32645843

[38] Fang R Wang C Jiang Q Lv M Gao P Yu X. NEMO-IKKbeta Are Essential for IRF3 and NF-kappaB Activation in the cGAS-STING Pathway. J Immunol (2017) 199(9):3222–33. doi: 10.4049/jimmunol.1700699 28939760

[39] Balka KR Louis C Saunders TL Smith AM Calleja DJ D’Silva DB. TBK1 and IKKepsilon Act Redundantly to Mediate STING-Induced NF-kappaB Responses in Myeloid Cells. Cell Rep (2020) 31(1):107492. doi: 10.1016/j.celrep.2020.03.056 32268090

[40] Wang Y Jia J Wang Y Li F Song X Qin S. Roles of HSV-1 Infection-Induced Microglial Immune Responses in CNS Diseases: Friends or Foes? Crit Rev Microbiol (2019) 45(5-6):581–94. doi: 10.1080/1040841X.2019.1660615 31512533

[41] Taniguchi T Takaoka A. The Interferon-Alpha/Beta System in Antiviral Responses: A Multimodal Machinery of Gene Regulation by the IRF Family of Transcription Factors. Curr Opin Immunol (2002) 14(1):111–6. doi: 10.1016/S0952-7915(01)00305-3 11790540

[42] Iwasaki A. A Virological View of Innate Immune Recognition. Annu Rev Microbiol (2012) 66:177–96. doi: 10.1146/annurev-micro-092611-150203 PMC3549330 22994491

[43] Andreeva L Hiller B Kostrewa D Lässig C de Oliveira Mann CC Jan Drexler D. cGAS Senses Long and HMGB/TFAM-Bound U-Turn DNA by Forming Protein-DNA Ladders. Nature (2017) 549(7672):394–8. doi: 10.1038/nature23890 28902841

[44] Herzner AM Hagmann CA Goldeck M Wolter S Kübler K Wittmann S. Sequence-Specific Activation of the DNA Sensor cGAS by Y-Form DNA Structures as Found in Primary HIV-1 cDNA. Nat Immunol (2015) 16(10):1025–33. doi: 10.1038/ni.3267 PMC4669199 26343537

[45] Du M Chen ZJ. DNA-Induced Liquid Phase Condensation of cGAS Activates Innate Immune Signaling. Science (2018) 361(6403):704–9. doi: 10.1126/science.aat1022 PMC9417938 29976794

[46] Kranzusch PJ Lee ASY Berger JM Doudna JA. Structure of Human cGAS Reveals a Conserved Family of Second-Messenger Enzymes in Innate Immunity. Cell Rep (2013) 3(5):1362–8. doi: 10.1016/j.celrep.2013.05.008 PMC3800681 23707061

[47] Luecke S Holleufer A Christensen MH Jonsson KL Boni GA Sorensen LK. cGAS Is Activated by DNA in a Length-Dependent Manner. EMBO Rep (2017) 18(10):1707–15. doi: 10.15252/embr.201744017 PMC5623850 28801534

[48] Gantier MP. Length Does Matter for cGAS. EMBO Rep (2017) 18(10):1675–6. doi: 10.15252/embr.201744773 PMC5623830 28827471

[49] Ni G Ma Z Damania B. cGAS and STING: At the Intersection of DNA and RNA Virus-Sensing Networks. PLoS Pathog (2018) 14(8):e1007148. doi: 10.1371/journal.ppat.1007148 30114241 PMC6095619

[50] West AP Khoury-Hanold W Staron M Tal MC Pineda CM Lang SM. Mitochondrial DNA Stress Primes the Antiviral Innate Immune Response. Nature (2015) 520(7548):553. doi: 10.1038/nature14156 PMC4409480 25642965

[51] Wang H Hu S Chen X Shi H Chen C Sun L. cGAS Is Essential for the Antitumor Effect of Immune Checkpoint Blockade. Proc Natl Acad Sci (2017) 114(7):1637–42. doi: 10.1073/pnas.1621363114 PMC5320994 28137885

[52] Moriyama M Koshiba T Ichinohe T. Influenza A Virus M2 Protein Triggers Mitochondrial DNA-Mediated Antiviral Immune Responses. Nat Commun (2019) 10:4624. doi: 10.1038/s41467-019-12632-5 31604929 PMC6789137

[53] Hopfner KP Hornung V. Molecular Mechanisms and Cellular Functions of cGAS-STING Signalling. Nat Rev Mol Cell Biol (2020) 21(9):501–21. doi: 10.1038/s41580-020-0244-x 32424334

[54] Barnett KC Coronas-Serna JM Zhou W Ernandes MJ Cao A Kranzusch PJ. Phosphoinositide Interactions Position cGAS at the Plasma Membrane to Ensure Efficient Distinction Between Self- and Viral DNA. Cell (2019) 176(6):1432–46.e11. doi: 10.1016/j.cell.2019.01.049 30827685 PMC6697112

[55] Orzalli MH Broekema NM Diner BA Hancks DC Elde NC Cristea IM. cGAS-Mediated Stabilization of IFI16 Promotes Innate Signaling During Herpes Simplex Virus Infection. Proc Natl Acad Sci U S A (2015) 112(14):E1773–81. doi: 10.1073/pnas.1424637112 PMC4394261 25831530

[56] Liu H Zhang H Wu X Ma D Wu J Wang L. Nuclear cGAS Suppresses DNA Repair and Promotes Tumorigenesis. Nature (2018) 563(7729):131–6. doi: 10.1038/s41586-018-0629-6 30356214

[57] Volkman HE Cambier S Gray EE Stetson DB. Tight Nuclear Tethering of cGAS Is Essential for Preventing Autoreactivity. Elife (2019) 8:e47491. doi: 10.7554/eLife.47491 31808743 PMC6927687

[58] Jiang H Xue X Panda S Kawale A Hooy RM Liang F. Chromatin-Bound cGAS Is an Inhibitor of DNA Repair and Hence Accelerates Genome Destabilization and Cell Death. EMBO J (2019) 38(21):e102718. doi: 10.15252/embj.2019102718 31544964 PMC6826206

[59] Michalski S de Oliveira Mann CC Stafford CA Witte G Bartho J Lammens K. Structural Basis for Sequestration and Autoinhibition of cGAS by Chromatin. Nature (2020) 587(7835):678–82. doi: 10.1038/s41586-020-2748-0 32911480

[60] Gentili M Lahaye X Nadalin F Nader GPF Lombardi EP Herve S. The N-Terminal Domain of cGAS Determines Preferential Association With Centromeric DNA and Innate Immune Activation in the Nucleus (Vol 26, pg 2377, 2019). Cell Rep (2019) 26(13):3474–. doi: 10.1016/j.celrep.2019.03.049 PMC6391843 30811988

[61] Zhao B Xu P Rowlett CM Jing T Shinde O Lei Y. The Molecular Basis of Tight Nuclear Tethering and Inactivation of cGAS. Nature (2020) 587(7835):673–7. doi: 10.1038/s41586-020-2749-z PMC7704945 32911481

[62] Pathare GR Decout A Glück S Cavadini S Makasheva K Hovius R. Structural Mechanism of cGAS Inhibition by the Nucleosome. Nature (2020) 587(7835):668–72. doi: 10.1038/s41586-020-2750-6 32911482

[63] Boyer JA Spangler CJ Strauss JD Cesmat AP Liu P McGinty RK. Structural Basis of Nucleosome-Dependent cGAS Inhibition. Science (2020) 370(6515):450–4. doi: 10.1126/science.abd0609 PMC8189757 32913000

[64] Kujirai T Zierhut C Takizawa Y Kim R Negishi L Uruma N. Structural Basis for the Inhibition of cGAS by Nucleosomes. Science (2020) 370(6515):455–8. doi: 10.1126/science.abd0237 PMC7584773 32912999

[65] Cao D Han X Fan X Xu RM Zhang X. Structural Basis for Nucleosome-Mediated Inhibition of cGAS Activity. Cell Res (2020) 30(12):1088–97. doi: 10.1038/s41422-020-00422-4 PMC7784699 33051594

[66] Guey B Wischnewski M Decout A Makasheva K Kaynak M Sakar MS. BAF Restricts cGAS on Nuclear DNA to Prevent Innate Immune Activation. Science (2020) 369(6505):823–8. doi: 10.1126/science.aaw6421 32792394

[67] Li T Huang T Du M Chen X Du F Ren J. Phosphorylation and Chromatin Tethering Prevent cGAS Activation During Mitosis. Science (2021) 371(6535):eabc5386. doi: 10.1126/science.abc5386 33542149 PMC8171060

[68] Gekara NO Jiang H. The Innate Immune DNA Sensor cGAS: A Membrane, Cytosolic, or Nuclear Protein? Sci Signal (2019) 12(581):eaax3521. doi: 10.1126/scisignal.aax3521 31088977

[69] Alharbi AS Garcin AJ Lennox KA Pradeloux S Wong C Straub S. Rational Design of Antisense Oligonucleotides Modulating the Activity of TLR7/8 Agonists. Nucleic Acids Res (2020) 48(13):7052–65. doi: 10.1093/nar/gkaa523 PMC7367172 32544249

[70] Valentin R Wong C Alharbi AS Pradeloux S Morros MP Lennox KA. Sequence-Dependent Inhibition of cGAS and TLR9 DNA Sensing by 2 ‘-O-Methyl Gapmer Oligonucleotides. Nucleic Acids Res (2021) 49(11):6082–99. doi: 10.1093/nar/gkab451 PMC8216285 34057477

[71] Xie W Lama L Adura C Tomita D Glickman JF Tuschl T. Human cGAS Catalytic Domain has an Additional DNA-Binding Interface That Enhances Enzymatic Activity and Liquid-Phase Condensation. P Natl Acad Sci U S A (2019) 116(24):11946–55. doi: 10.1073/pnas.1905013116 PMC6575157 31142647

[72] Feijo Delgado F Cermak N Hecht VC Son S Li Y Knudsen SM. Intracellular Water Exchange for Measuring the Dry Mass, Water Mass and Changes in Chemical Composition of Living Cells. PLoS One (2013) 8(7):e67590. doi: 10.1371/journal.pone.0067590 23844039 PMC3699654

[73] Palazzo AF Lee ES. Non-Coding RNA: What Is Functional and What Is Junk? Front Genet (2015) 6:2. doi: 10.3389/fgene.2015.00002 25674102 PMC4306305

[74] Frenkel-Morgenstern M Danon T Christian T Igarashi T Cohen L Hou YM. Genes Adopt Non-Optimal Codon Usage to Generate Cell Cycle-Dependent Oscillations in Protein Levels. Mol Syst Biol (2012) 8:572. doi: 10.1038/msb.2012.3 22373820 PMC3293633

[75] Russo J Russo IH. Techniques and Methodological Approaches in Breast Cancer Research. Springer (2014). pp. 287.

[76] Karalyan ZA Izmailyan RA Abroyan LO Avetisyan AS Hakobyan LA Zakaryan HS. Evaluation of Viral Genome Copies Within Viral Factories on Different DNA Viruses. J Histochem Cytochem (2018) 66(5):359–65. doi: 10.1369/0022155417749490 PMC5958354 29298122

[77] Chen LL. The Biogenesis and Emerging Roles of Circular RNAs. Nat Rev Mol Cell Bio (2016) 17(4):205–11. doi: 10.1038/nrm.2015.32 26908011

[78] Shang QF Yang Z Jia RB Ge SF. The Novel Roles of circRNAs in Human Cancer. Mol Cancer (2019) 18:6. doi: 10.1186/s12943-018-0934-6 30626395 PMC6325800

[79] Steinhagen F Zillinger T Peukert K Fox M Thudium M Barchet W. Suppressive Oligodeoxynucleotides Containing TTAGGG Motifs Inhibit cGAS Activation in Human Monocytes. Eur J Immunol (2018) 48(4):605–11. doi: 10.1002/eji.201747338 PMC6386451 29215161

[80] Mankan AK Schmidt T Chauhan D Goldeck M Höning K Gaidt M. Cytosolic RNA : DNA Hybrids Activate the cGAS-STING Axis. EMBO J (2014) 33(24):2937–46. doi: 10.15252/embj.201488726 PMC4282641 25425575

[81] Prichard MN Jairath S Penfold ME St Jeor S Bohlman MC Pari GS. Identification of Persistent RNA-DNA Hybrid Structures Within the Origin of Replication of Human Cytomegalovirus. J Virol (1998) 72(9):6997–7004. doi: 10.1128/JVI.72.9.6997-7004.1998 9696791 PMC109919

[82] Rennekamp AJ Lieberman PM. Initiation of Epstein-Barr Virus Lytic Replication Requires Transcription and the Formation of a Stable RNA-DNA Hybrid Molecule at OriLyt. J Virol (2011) 85(6):2837–50. doi: 10.1128/JVI.02175-10 PMC3067963 21191028

[83] Telesnitsky A Goff SP. “Reverse Transcriptase and the Generation of Retroviral DNA”. In: Coffin JM Hughes SH Varmus HE, editors. Retroviruses. Cold Spring Harbor (NY: Cold Spring Harbor Laboratory Press (1997). 21433342

[84] Morchikh M Cribier A Raffel R Amraoui S Cau J Severac D. HEXIM1 and NEAT1 Long Non-Coding RNA Form a Multi-Subunit Complex That Regulates DNA-Mediated Innate Immune Response. Mol Cell (2017) 67(3):387–99.e5. doi: 10.1016/j.molcel.2017.06.020 28712728

[85] Sidibé H Dubinski A Vande Velde C. The Multi-Functional RNA-Binding Protein G3BP1 and Its Potential Implication in Neurodegenerative Disease. J Neurochem (2020) 157(4):944–62. doi: 10.1111/jnc.15280 PMC8248322 33349931

[86] van Gent M Sparrer KMJ Gack MU. TRIM Proteins and Their Roles in Antiviral Host Defenses. Annu Rev Virol (2018) 5(1):385–405. doi: 10.1146/annurev-virology-092917-043323 29949725 PMC6186430

[87] Goyani S Roy M Singh R. TRIM-NHL as RNA Binding Ubiquitin E3 Ligase (RBUL): Implication in Development and Disease Pathogenesis. Bba-Mol Basis Dis (2021) 1687(7):166066. doi: 10.1016/j.bbadis.2020.166066 33418035

[88] Thapa P Shanmugam N Pokrzywa W. Ubiquitin Signaling Regulates RNA Biogenesis, Processing, and Metabolism. Bioessays (2020) 42(1):e1900171. doi: 10.1002/bies.201900171 31778250

[89] Yang D Li NL Wei D Liu B Guo F Elbahesh H. The E3 Ligase TRIM56 Is a Host Restriction Factor of Zika Virus and Depends on Its RNA-Binding Activity But Not miRNA Regulation, for Antiviral Function. PLoS Negl Trop Dis (2019) 13(6):e0007537. doi: 10.1371/journal.pntd.0007537 31251739 PMC6623546

[90] Chen M Meng Q Qin Y Liang P Tan P He L. TRIM14 Inhibits cGAS Degradation Mediated by Selective Autophagy Receptor P62 to Promote Innate Immune Responses. Mol Cell (2016) 64(1):105–19. doi: 10.1016/j.molcel.2016.08.025 27666593

[91] Williams FP Haubrich K Perez-Borrajero C Hennig J. Emerging RNA-Binding Roles in the TRIM Family of Ubiquitin Ligases. Biol Chem (2019) 400(11):1443–64. doi: 10.1515/hsz-2019-0158 31120853

[92] Lian H Zang R Wei J Ye W Hu MM Chen YD. The Zinc-Finger Protein ZCCHC3 Binds RNA and Facilitates Viral RNA Sensing and Activation of the RIG-I-Like Receptors. Immunity (2018) 49(3):438–48.e5. doi: 10.1016/j.immuni.2018.08.014 30193849

[93] Liao CY Lei CQ Shu HB. PCBP1 Modulates the Innate Immune Response by Facilitating the Binding of cGAS to DNA. Cell Mol Immunol (2020) 18(10):2334–43. doi: 10.1038/s41423-020-0462-3 PMC8484664 32415261

[94] Lian H Wei J Zang R Ye W Yang Q Zhang XN. ZCCHC3 Is a Co-Sensor of cGAS for dsDNA Recognition in Innate Immune Response. Nat Commun (2018) 9(1):3349. doi: 10.1038/s41467-018-05559-w 30135424 PMC6105683

[95] Zang R Lian H Zhong X Yang Q Shu HB. ZCCHC3 Modulates TLR3-Mediated Signaling by Promoting Recruitment of TRIF to TLR3. J Mol Cell Biol (2020) 12(4):251–62. doi: 10.1093/jmcb/mjaa004 PMC7232131 32133501

[96] Yang P Mathieu C Kolaitis RM Zhang P Messing J Yurtsever U. G3BP1 Is a Tunable Switch That Triggers Phase Separation to Assemble Stress Granules. Cell (2020) 181(2):325–45.e28. doi: 10.1016/j.cell.2020.03.046 32302571 PMC7448383

[97] Sanders DW Kedersha N Lee DSW Strom AR Drake V Riback JA. Competing Protein-RNA Interaction Networks Control Multiphase Intracellular Organization. Cell (2020) 181(2):306–24.e28. doi: 10.1016/j.cell.2020.03.050 32302570 PMC7816278

[98] Guillen-Boixet J Kopach A Holehouse AS Wittmann S Jahnel M Schlussler R. RNA-Induced Conformational Switching and Clustering of G3BP Drive Stress Granule Assembly by Condensation. Cell (2020) 181(2):346–61.e17. doi: 10.1016/j.cell.2020.03.049 32302572 PMC7181197

[99] Liu ZS Cai H Xue W Wang M Xia T Li WJ. G3BP1 Promotes DNA Binding and Activation of cGAS. Nat Immunol (2019) 20(1):18–28. doi: 10.1038/s41590-018-0262-4 30510222 PMC8276115

[100] Eiermann N Haneke K Sun Z Stoecklin G Ruggieri A. Dance With the Devil: Stress Granules and Signaling in Antiviral Responses. Viruses (2020) 12(9):984. doi: 10.3390/v12090984 PMC7552005 32899736

[101] Knott GJ Bond CS Fox AH. The DBHS Proteins SFPQ, NONO and PSPC1: A Multipurpose Molecular Scaffold. Nucleic Acids Res (2016) 44(9):3989–4004. doi: 10.1093/nar/gkw271 27084935 PMC4872119

[102] Lahaye X Gentili M Silvin A Conrad C Picard L Jouve M. NONO Detects the Nuclear HIV Capsid to Promote cGAS-Mediated Innate Immune Activation. Cell (2018) 175(2):488–501.e22. doi: 10.1016/j.cell.2018.08.062 30270045

[103] Wan L Juszkiewicz S Blears D Bajpe PK Han Z Faull P. Translation Stress and Collided Ribosomes Are Co-Activators of cGAS. Mol Cell (2021) 81(13):2808–22.e10. doi: 10.1016/j.molcel.2021.05.018 34111399 PMC8260207

[104] Anantharaman V Koonin EV Aravind L. Comparative Genomics and Evolution of Proteins Involved in RNA Metabolism. Nucleic Acids Res (2002) 30(7):1427–64. doi: 10.1093/nar/30.7.1427 PMC101826 11917006

[105] Gerstberger S Hafner M Tuschl T. A Census of Human RNA-Binding Proteins. Nat Rev Genet (2014) 15(12):829–45. doi: 10.1038/nrg3813 PMC11148870 25365966

[106] Corley M Burns MC Yeo GW. How RNA-Binding Proteins Interact With RNA: Molecules and Mechanisms. Mol Cell (2020) 78(1):9–29. doi: 10.1016/j.molcel.2020.03.011 32243832 PMC7202378

[107] Albihlal WS Gerber AP. Unconventional RNA-Binding Proteins: An Uncharted Zone in RNA Biology. FEBS Lett (2018) 592(17):2917–31. doi: 10.1002/1873-3468.13161 29908064

[108] Ramanathan M Porter DF Khavari PA. Methods to Study RNA-Protein Interactions. Nat Methods (2019) 16(3):225–34. doi: 10.1038/s41592-019-0330-1 PMC6692137 30804549

[109] Hentze MW Castello A Schwarzl T Preiss T. A Brave New World of RNA-Binding Proteins. Nat Rev Mol Cell Biol (2018) 19(5):327–41. doi: 10.1038/nrm.2017.130 29339797

[110] Ablasser A Chen ZJ. cGAS in Action: Expanding Roles in Immunity and Inflammation. Science (2019) 363(6431):1055–+. doi: 10.1126/science.aat8657 30846571

[111] Hornung V Hartmann R Ablasser A Hopfner KP. OAS Proteins and cGAS: Unifying Concepts in Sensing and Responding to Cytosolic Nucleic Acids. Nat Rev Immunol (2014) 14(8):521–8. doi: 10.1038/nri3719 PMC7097587 25033909

[112] Cadena C Hur S. Filament-Like Assemblies of Intracellular Nucleic Acid Sensors: Commonalities and Differences. Mol Cell (2019) 76(2):243–54. doi: 10.1016/j.molcel.2019.09.023 PMC6880955 31626748

[113] Slavik KM Morehouse BR Ragucci AE Zhou W Ai XL Chen YQ. cGAS-Like Receptors Sense RNA and Control 3 ‘ 2 ‘-cGAMP Signalling in Drosophila. Nature (2021) 597(7874):109. doi: 10.1038/s41586-021-03743-5 PMC8410604 34261127

[114] Holleufer A Winther KG Gad HH Ai XL Chen YQ Li LH. Two cGAS-Like Receptors Induce Antiviral Immunity in Drosophila. Nature (2021) 597(7874):114. doi: 10.1038/s41586-021-03800-z 34261128

[115] Bellucci M Agostini F Masin M Tartaglia GG. Predicting Protein Associations With Long Noncoding RNAs. Nat Methods (2011) 8(6):444–5. doi: 10.1038/nmeth.1611 21623348

[116] Lang B Armaos A Tartaglia GG. RNAct: Protein-RNA Interaction Predictions for Model Organisms With Supporting Experimental Data. Nucleic Acids Res (2019) 47(D1):D601–D6. doi: 10.1093/nar/gky967 PMC6324028 30445601

[117] Colantoni A Rupert J Vandelli A Tartaglia GG Zacco E. Zooming in on Protein-RNA Interactions: A Multi-Level Workflow to Identify Interaction Partners. Biochem Soc Trans (2020) 48(4):1529–43. doi: 10.1042/BST20191059 PMC7458403 32820806

[118] Yang W Zhou J Zhang K Li L Xu Y Ma K. Identification and Validation of the Clinical Roles of the VHL-Related LncRNAs in Clear Cell Renal Cell Carcinoma. J Cancer (2021) 12(9):2702–14. doi: 10.7150/jca.55113 PMC8040721 33854630

[119] Ribeiro DM Zanzoni A Cipriano A Delli Ponti R Spinelli L Ballarino M. Protein Complex Scaffolding Predicted as a Prevalent Function of Long Non-Coding RNAs. Nucleic Acids Res (2018) 46(2):917–28. doi: 10.1093/nar/gkx1169 PMC5778612 29165713

[120] Wang Y Luo W Huang L Xiao J Song X Li F. A Novel lncRNA Linc-AhRA Negatively Regulates Innate Antiviral Response in Murine Microglia Upon Neurotropic Herpesvirus Infection. Theranostics (2021) 11(19):9623–51. doi: 10.7150/thno.64880 PMC8490526 34646390

[121] Livi CM Klus P Delli Ponti R Tartaglia GG. catRAPID Signature: Identification of Ribonucleoproteins and RNA-Binding Regions. Bioinformatics (2016) 32(5):773–5. doi: 10.1093/bioinformatics/btv629 PMC4795616 26520853

[122] Hartmann R Justesen J Sarkar SN Sen GC Yee VC. Crystal Structure of the 2'-Specific and Double-Stranded RNA-Activated Interferon-Induced Antiviral Protein 2'-5'-Oligoadenylate Synthetase. Mol Cell (2003) 12(5):1173–85. 10.1016/s1097-2765(03)00433-7 14636576

[123] Agostini F Zanzoni A Klus P Marchese D Cirillo D Tartaglia GG. catRAPID Omics: A Web Server for Large-Scale Prediction of Protein-RNA Interactions. Bioinformatics (2013) 29(22):2928–30. doi: 10.1093/bioinformatics/btt495 PMC3810848 23975767

[124] Armaos A Colantoni A Proietti G Rupert J Tartaglia GG. catRAPID Omics V2.0: Going Deeper and Wider in the Prediction of Protein-RNA Interactions. Nucleic Acids Res (2021) 49(W1):W72–9. doi: 10.1093/nar/gkab393 PMC8262727 34086933

[125] Ferre F Colantoni A Helmer-Citterich M. Revealing protein-lncRNA Interaction. Brief Bioinform (2016) 17(1):106–16. doi: 10.1093/bib/bbv031 PMC4719072 26041786

[126] Xu J Wang Z Jin X Li L Pan T. Methods for Identification of Protein-RNA Interaction. Adv Exp Med Biol (2018) 1094:117–26. doi: 10.1007/978-981-13-0719-5_12 30191493

[127] Hellman LM Fried MG. Electrophoretic Mobility Shift Assay (EMSA) for Detecting Protein-Nucleic Acid Interactions. Nat Protoc (2007) 2(8):1849–61. doi: 10.1038/nprot.2007.249 PMC2757439 17703195

[128] Bai Q Bai Z Sun L. Detection of RNA-Binding Proteins by *In Vitro* RNA Pull-Down in Adipocyte Culture. J Vis Exp (2016) 113):e54207. doi: 10.3791/54207 27500988

[129] Wang W Caldwell MC Lin S Furneaux H Gorospe M. HuR Regulates Cyclin A and Cyclin B1 mRNA Stability During Cell Proliferation. EMBO J (2000) 19(10):2340–50. doi: 10.1093/emboj/19.10.2340 PMC384372 10811625

[130] Tuerk C Gold L. Systematic Evolution of Ligands by Exponential Enrichment: RNA Ligands to Bacteriophage T4 DNA Polymerase. Science (1990) 249(4968):505–10. doi: 10.1126/science.2200121 2200121

[131] Lorenz C von Pelchrzim F Schroeder R. Genomic Systematic Evolution of Ligands by Exponential Enrichment (Genomic SELEX) for the Identification of Protein-Binding RNAs Independent of Their Expression Levels. Nat Protoc (2006) 1(5):2204–12. doi: 10.1038/nprot.2006.372 17406458

[132] Stoltenburg R Reinemann C Strehlitz B. SELEX–a (R)Evolutionary Method to Generate High-Affinity Nucleic Acid Ligands. Biomol Eng (2007) 24(4):381–403. doi: 10.1016/j.bioeng.2007.06.001 17627883

[133] Ray D Kazan H Chan ET Pena Castillo L Chaudhry S Talukder S. Rapid and Systematic Analysis of the RNA Recognition Specificities of RNA-Binding Proteins. Nat Biotechnol (2009) 27(7):667–70. doi: 10.1038/nbt.1550 19561594

[134] Lambert N Robertson A Jangi M McGeary S Sharp PA Burge CB. RNA Bind-N-Seq: Quantitative Assessment of the Sequence and Structural Binding Specificity of RNA Binding Proteins. Mol Cell (2014) 54(5):887–900. doi: 10.1016/j.molcel.2014.04.016 24837674 PMC4142047

[135] Lambert NJ Robertson AD Burge CB. RNA Bind-N-Seq: Measuring the Binding Affinity Landscape of RNA-Binding Proteins. Methods Enzymol (2015) 558:465–93. doi: 10.1016/bs.mie.2015.02.007 PMC5576890 26068750

[136] Gagliardi M Matarazzo MR. RIP: RNA Immunoprecipitation. Methods Mol Biol (2016) 1480:73–86. doi: 10.1007/978-1-4939-6380-5_7 27659976

[137] Ule J Jensen K Mele A Darnell RB. CLIP: A Method for Identifying Protein-RNA Interaction Sites in Living Cells. Methods (2005) 37(4):376–86. doi: 10.1016/j.ymeth.2005.07.018 16314267

[138] Foley SW Gregory BD. Protein Interaction Profile Sequencing (PIP-Seq). Curr Protoc Mol Biol (2016) 116:27 5 1– 5 15. doi: 10.1002/cpmb.21 PMC5762119 27723083

[139] Carpenter S Aiello D Atianand MK Ricci EP Gandhi P Hall LL. A Long Noncoding RNA Mediates Both Activation and Repression of Immune Response Genes. Science (2013) 341(6147):789–92. doi: 10.1126/science.1240925 PMC4376668 23907535

[140] Kazan H Ray D Chan ET Hughes TR Morris Q. RNAcontext: A New Method for Learning the Sequence and Structure Binding Preferences of RNA-Binding Proteins. PLoS Comput Biol (2010) 6(7):e1000832. doi: 10.1371/journal.pcbi.1000832 20617199 PMC2895634

[141] Suresh V Liu L Adjeroh D Zhou X. RPI-Pred: Predicting ncRNA-Protein Interaction Using Sequence and Structural Information. Nucleic Acids Res (2015) 43(3):1370–9. doi: 10.1093/nar/gkv020 PMC4330382 25609700

[142] Cheng ZZ Zhou SG Guan JH. Computationally Predicting Protein-RNA Interactions Using Only Positive and Unlabeled Examples. J Bioinf Comput Biol (2015) 13(3):1541005. doi: 10.1142/S021972001541005X 25790785

[143] Muppirala UK Honavar VG Dobbs D. Predicting RNA-Protein Interactions Using Only Sequence Information. BMC Bioinf (2011) 12:489. doi: 10.1186/1471-2105-12-489 PMC3322362 22192482

[144] Ankö ML Neugebauer KM. RNA-Protein Interactions *In Vivo*: Global Gets Specific. Trends Biochem Sci (2012) 37(7):255–62. doi: 10.1016/j.tibs.2012.02.005 22425269

[145] Hogan DJ Riordan DP Gerber AP Herschlag D Brown PO. Diverse RNA-Binding Proteins Interact With Functionally Related Sets of RNAs, Suggesting an Extensive Regulatory System. PLoS Biol (2008) 6(10):e255. doi: 10.1371/journal.pbio.0060255 18959479 PMC2573929

[146] Hu H Zhu C Ai H Zhang L Zhao J Zhao Q. LPI-ETSLP: lncRNA-Protein Interaction Prediction Using Eigenvalue Transformation-Based Semi-Supervised Link Prediction. Mol Biosyst (2017) 13(9):1781–7. doi: 10.1039/C7MB00290D 28702594

[147] Zhao Q Zhang Y Hu H Ren G Zhang W Liu H. IRWNRLPI: Integrating Random Walk and Neighborhood Regularized Logistic Matrix Factorization for lncRNA-Protein Interaction Prediction. Front Genet (2018) 9:239. doi: 10.3389/fgene.2018.00239 30023002 PMC6040094

[148] Kloetgen A Munch PC Borkhardt A Hoell JI McHardy AC. Corrigendum to: Biochemical and Bioinformatic Methods for Elucidating the Role of RNA-Protein Interactions in Posttranscriptional Regulation. Brief Funct Genomics (2019) 18(6):433. doi: 10.1093/bfgp/elz014 31169895 PMC6920523

[149] Pan X Yang Y Xia CQ Mirza AH Shen HB. Recent Methodology Progress of Deep Learning for RNA-Protein Interaction Prediction. Wiley Interdiscip Rev RNA (2019) 10(6):e1544. doi: 10.1002/wrna.1544 31067608

[150] Tan X Sun L Chen J Chen ZJ. Detection of Microbial Infections Through Innate Immune Sensing of Nucleic Acids. Annu Rev Microbiol (2018) 72:447–78. doi: 10.1146/annurev-micro-102215-095605 30200854

[151] Gray EE Treuting PM Woodward JJ Stetson DB. Cutting Edge: cGAS Is Required for Lethal Autoimmune Disease in the Trex1-Deficient Mouse Model of Aicardi-Goutieres Syndrome. J Immunol (2015) 195(5):1939–43. doi: 10.4049/jimmunol.1500969 PMC4546858 26223655

